# Suitability of drone olfactory sensitivity as a selection trait for *Varroa*-resistance in honeybees

**DOI:** 10.1038/s41598-021-97191-w

**Published:** 2021-09-06

**Authors:** Ivelina Ivanova, Kaspar Bienefeld

**Affiliations:** 1 Institute for Bee Research Hohen Neuendorf, Friedrich-Engels-Str. 32, 16540 Hohen Neuendorf, Germany; 2 Institute of Parasitology and Tropical Veterinary Medicine, Faculty of Veterinary Medicine, Free University of Berlin, Robert-von-Ostertag-Str. 7-13, Building 35, 14163 Berlin, Germany

**Keywords:** Agricultural genetics, Animal breeding, Behavioural genetics

## Abstract

The most effective strategy against brood diseases, such as those stemming from infestation by the mite *Varroa destructor,* is the early detection and removal of sick brood. Recent findings suggest that genes associated with worker bee olfactory perception play a central role in *Varroa*-sensitive hygiene (VSH). In this study, the odour sensitivity of *Apis mellifera* drones was examined through proboscis extension response (PER) conditioning. Individuals sensitive/insensitive to the two *Varroa*-parasitised-brood odours (*extract-low* and *extract-high*) were used for breeding. Twenty-one queens from a VSH-selected line (*SelQ*) and nineteen queens from a nonselected line (*ConQ*) were single-drone-inseminated with sperm from drones that showed either sensitivity (*SenD*+) or insensitivity (*SenD−*) to the two extracts. Individual VSH behaviour in a total of 5072 offspring of these combinations (*SelQ × SenD*+, *SelQ* *× SenD−*, *ConQ × SenD*+, *ConQ × SenD−*) was subsequently observed in a specially designed observation unit with infrared light. The results from the video observation were also separately examined, considering the genetic origin (VSH-selected or nonselected line) of the participating queens and drones. While the drone PER conditioning results were not significantly reflected in the VSH results of the respective offspring, the genetic origin of the participating queens/drones was crucial for VSH manifestation.

## Introduction

The ectoparasitic mite *Varroa destructor* plays a dominant role in colony losses of the European honeybee *Apis mellifera*^1–3^. Currently, available treatments for *Varroa*-infested colonies such as pyrethroids and formic acid are not only labour intensive but also leave residues in honeybee products^4,5^. In addition, studies have shown an alarming tendency of increasing mite resistance against miticides^6–8^. While current treatment methods provide only temporary benefits, breeding colonies resistant to *V. destructor* is considered the only long-term solution^9,10^.

The antennae of bees play an essential role in perceiving their environment and communication within the hive^11,12^. Amid this process, both olfactory and tactile stimuli are perceived and processed. One of the natural defence mechanisms of honeybees that has proven effective against *V. destructor* is hygienic behaviour. Mechanisms similar to the hygienic behaviour of honeybees have also been observed in other social insects^13–15^. In honeybees, hygienic behaviour consists of detecting, uncapping, and removing damaged brood^16–18^. This particular behaviour directed towards *V. destructor* has received different names during the years^19–23^. Among others, the term "suppression of mite reproduction" (SMR) was created by Harbo and Harris^15^ to describe the lack of viable progeny of the mite observed in resistant colonies during their experiments. Subsequently, SMR was renamed *Varroa*-sensitive hygiene (VSH), as the observed suppression was found to be the result of removing reproductive mites and not of inhibiting reproduction of *V. destructor* in resistant colonies^21,24,25^.

Research has shown that selective breeding can improve the colonies' performance regarding their hygienic behaviour against *V. destructor*^9,21,26,27^. VSH is assumed to be based on the differential expression of genes responsible for the olfactory system and perception^28–32^. Mondet et al*.*^33^ presented evidence that all worker bees can detect *Varroa*-parasitisation-specific compounds, but only bees performing VSH can distinguish those from the healthy brood odour.

Signals from the damaged brood are present on the cell cap^34^. VSH bees use their olfaction to perceive these cues emitted by the infested pupae and thereby target the most compromised brood cells containing multiple mature females and higher numbers of mite progeny^35,36^. Through typical movements with the head, the worker bees can localize the damaged brood very accurately^37^. By uncapping and removing the diseased brood, VSH bees diminish the mite's spread in the colony^38^. In some cases, instead of removing the parasitised brood, workers open and recap parasitised brood cells multiple times. This behaviour disrupts mite reproduction without sacrificing the developing brood^39,40^.

Differences in the odour discrimination abilities of hygienic and nonhygienic colonies have also been observed under laboratory conditions^41,42^. Masterman et al*.*^41^ used differential conditioning with two odour combinations—geraniol/1-hexanal and odour of healthy pupae/odour of chalkbrood infested pupae—to examine the discrimination abilities of worker bees from hygienic and nonhygienic lines. While there was no significant difference between the two genetic lines when presented with flower odours, Masterman et al*.*^41^ observed discrepancies in the perception of the brood odour. The hygienic line discriminated better between the two brood odours during the conditioning process than did the nonhygienic line. The authors suspected a genetically induced increased specific odour sensitivity to pathogens in the hygienic line, which would allow worker bees to remove sick individuals from the population more efficiently. Masterman et al*.*^41^ used conditioning with the so-called proboscis extension response (PER).

The PER is a biological reflex that occurs in different species of insects due to antennal stimulation^43^. Honeybees usually exhibit this behaviour while foraging or during trophallaxis. PER is easily replicated under laboratory conditions. Based on Pavlovian classical conditioning, conditioning using PER was first introduced by K. Takeda in 1961 and has been used as a foundation for many olfactory experiments ever since^43–48^. Among others, the PER conditioning is widely applied for observing the learning ability of individual honeybees^46,48^, the odour sensitivity connected to VSH^33,42^ and the adverse effects of pesticides on honeybee behaviour^49,50^. The subject learns to associate a conditioned stimulus (CS)—usually an odour—with an unconditioned stimulus (US) such as a sugar solution^51^. The odour presentation leads to the extension of the mouthparts (proboscis), as a reward is expected. Through varying concentrations of the odour substance, the individual animal's odour sensitivity and perception threshold can be determined^52–54^.

While current breeding strategies concentrate on worker bees and their ability to recognize mite-infested cells, our focus lies in identifying the drone's role as a genetic carrier for the manifestation of VSH. Because of drones’ impressive ability to detect the queen from a distance during mating flights using olfactory cues^55^, we speculated that the use of individually tested drones could be a very efficient approach to significantly improve the genetic progress in developing *Varroa*-resistance. Drones are haploid, and all their genetic material is completely passed on to the offspring without the Mendelian sampling effect. Having this in mind, we used PER conditioning to noninvasively evaluate the drone’s odour sensitivity towards an extract of *Varroa*-parasitised brood. Unlike other brood diseases, such as chalkbrood, which cause more extensive damage to the brood, the signals emitted from the *Varroa*-parasitised brood are much weaker^31^. The perception of the subtle stimulus caused by the parasitisation with *V. destructor* is therefore suitable for selecting for a better resistance not only against *V. destructor* but also against most brood diseases.

To observe whether brood odour sensitivity would be reflected in the VSH of the F1 generation, queens underwent a one-drone insemination, and the offspring of the tested drones (worker bees) was observed in a unit with infrared light for its ability to detect and remove artificially *Varroa*-infested brood.

## Results

### Drone conditioning of the two lines regarding different odour concentrations

The selection of drones for artificial insemination was performed through two conditioning experiments using different concentrations of a *Varroa*-parasitised pupae extract—*extract-low* and *extract-high*. The solvent used for the creation of the extract was used as CS−. During the conditioning, the drones had to differentiate between the *Varroa*-parasitised brood odour and the solvent control. The conditioning consisted of six trials (CS+, CS−, CS−, CS+, CS+, CS−) and was followed by an unrewarded presentation of both stimuli (CS+ and CS−). Drones from a line selected for VSH and drones from a nonselected line were conditioned with one of the two extracts (*high* and *low*).

Before the start of the main experiment, preliminary tests were conducted in order to determine which odour concentrations were suitable for our experimental design. An important criterion for the decision was to obtain a sufficient number of successfully conditioned drones for the sperm extraction. After a series of preliminary tests, the concentrations of *extracts high* and *low* were deemed suitable for the official experiment. A third of the drones (30%) managed to perceive *extract-low.* For *extract-high*, that number was ~ 60%.

**Figure 1:**
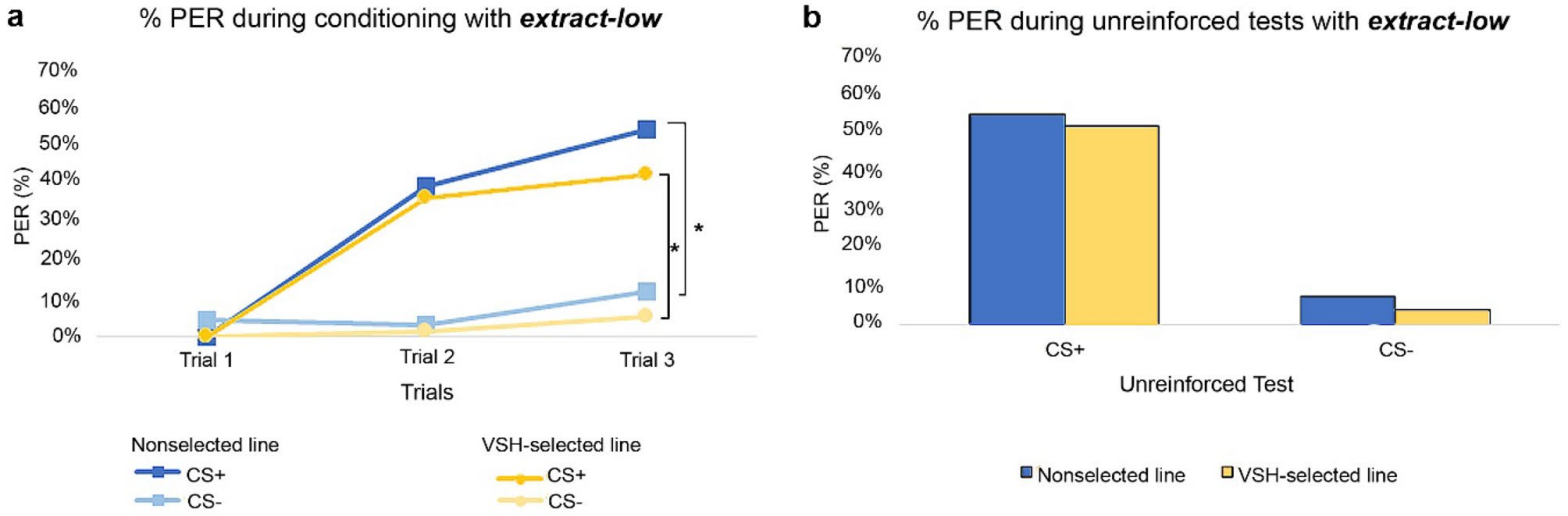
Drone performance during PER-conditioning experiment with *extract-low*. Acquisition (**a**) and results from the unreinforced tests (**b**) are shown for both stimuli (CS+ and CS−) and origins (nonselected line, VSH-selected line). The curves display the behavioural reaction—proboscis extension—for the reinforced (CS+) and the non-reinforced (CS−) stimulus. The bars show the behavioural reaction during the unreinforced tests with both stimuli. A total of 223 drones were tested using *extract-low*. The stimulus effect (reinforced, non-reinforced) was significant—(*) p < 0.001.

**Figure 2:**
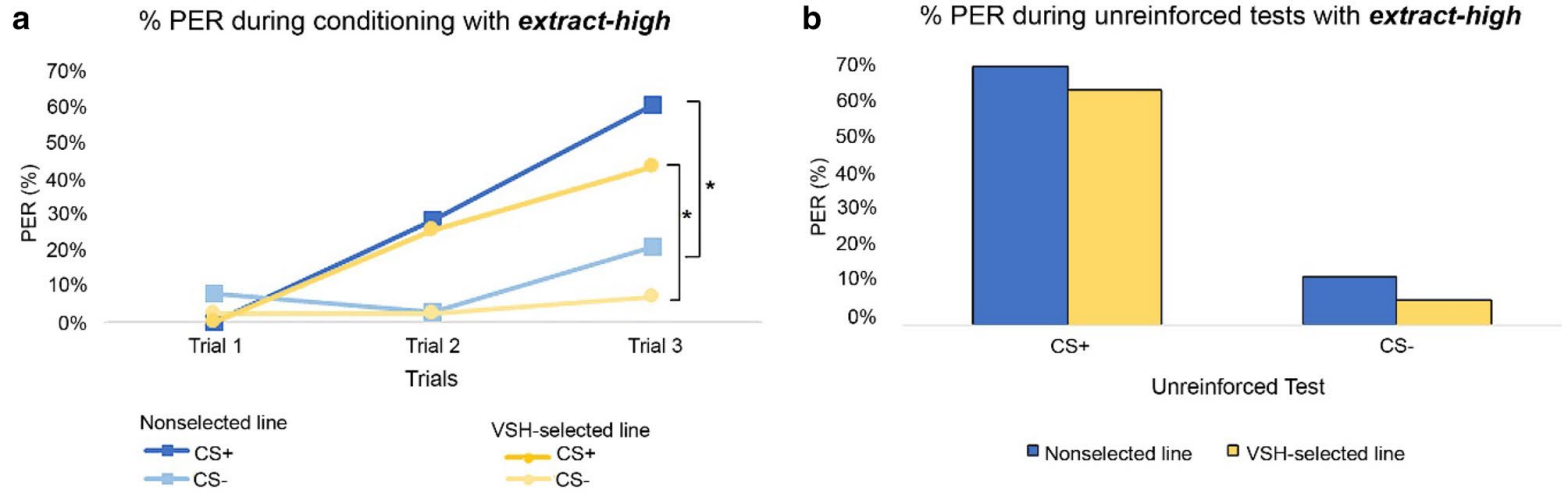
Drone performance during PER-conditioning experiment with *extract-high*. Acquisition (**a**) and results from the unreinforced tests (**b**) are shown for both stimuli (CS+ and CS−) and origins (nonselected line, VSH-selected line). The curves display the behavioural reaction—proboscis extension—for the reinforced (CS+) and the non-reinforced (CS−) stimulus. The bars show the behavioural reaction during the unreinforced tests with both stimuli. A total of 202 drones were tested using *extract-high*. The stimulus effect (reinforced, non-reinforced) was significant—(*) p < 0.001.

During the main experiment drones conditioned with both extracts exhibited an increase in the behavioural reactions (proboscis extension) when the CS+ was paired with the reward. This was not the case with CS−. The responses to CS− remained almost constant (Figs. 1, 2).

The number of drones successfully conditioned to *extract-low* and *extract-high* was 39% and 46%, respectively. During the main experiment, the drones excluded for not responding to the sugar stimulus amounted to 22% for *extract-low* and 16% for *extract-high.*

To evaluate whether the origin of the drones participating in the conditioning played a role in the conditioning outcome, the results of the two lines were analysed separately using a Generalized linear mixed model (GLMM). The drones from the nonselected line were set as reference group in the model and the stimulus effect (CS+ or CS−) was also included. The temperature during the conditioning and the drone’s mother were used as random factors in the model.

The GLMM model showed no statistically significant difference between the conditioning results of the VSH-selected and nonselected line drones. These findings applied to both *extract-low* (GLMM, p = 0.36; CI − 0.80; 0.29) and *extract-high* (GLMM, p = 0.14; CI − 0.14; 0.96). The stimulus effect proved to be significant (*extract-low*: GLMM, p < 0.001; CI 2.34; 3.75 and *extract-high*: GLMM, p < 0.001; CI 2.32; 3.54), showing a conditioning success only for the rewarded stimulus CS+ but not for the unrewarded CS− (Figs. 1, 2). The temperature during the conditioning and the drone’s mother had no significant effect on the conditioning results for either *extract-high* or *extract*-*low*.

### Mating design

Depending on the conditioning results, the drones were divided into two groups—“*Varroa*-parasitised-brood-odour sensitive” (*SenD*+) and “*Varroa*-parasitised-brood-odour insensitive” (*SenD−*). "Sensitive" drones responded to the CS+ but not the CS− during the last two trials and during the unrewarded tests with *extract-low*—Trials: CS+, CS−, CS−, CS+, CS+, CS−; Unrewarded test: CS+, CS−. The "insensitive" drones responded to the US throughout the experiment with *extract-high* but showed no positive responses to the CS+ during the last two trials, and the unrewarded tests indicated a negative conditioning outcome.

Queens from both a VSH-selected line and a nonselected line were one-drone inseminated with sperm from the “sensitive” or “insensitive” drones. Four groups were created during the one-drone insemination depending on the queen's affiliation with the VSH-selected line (*SelQ*) or nonselected line (*ConQ*)^56^ and the drone's olfactory sensitivity towards the *Varroa*-parasitised-brood odour.

The groups were created without regards to the genetic origin of the drones. Because drones from both origins were tested during the experiment, each group consisted of queens inseminated with sperm from drones from both lines (Suppl. Table 1).

Of the 87 *Varroa*-parasitised-brood-odour sensitive drones that qualified for insemination, only 26 were used for the insemination of queens since the rest did not have sperm. Of those 26, only 20 queens produced enough offspring to participate further in the experiment.

Of the 48 *Varroa*-parasitised-brood-odour insensitive drones, 22 were used for insemination. Of those, 20 had enough offspring to participate in the experiment.

### Video observation

The offspring (worker bees) of the one-drone inseminated queens was marked with numbered plates on the dorsal thorax and its VSH towards an artificially *Varroa* mite-infested brood frame was recorded during six days in an infrared video observation unit. The video observation was performed three times (courses) with different bees during the experiment.

For the evaluation of the video recording two activities were of importance. Beginner bees were the first to open a mite infested cell. Helper bees enlarged the hole in the cell cap created by the beginner. If the cell was resealed, the next beginner and helper bees were noted.

#### VSH of groups considering drones’ olfactory sensitivity in PER conditioning experiment

The new generation of worker bees was divided into four groups considering their mother’s origin (VSH-selected line *SelQ* or nonselected line *ConQ*) and their father’s odour sensitivity—*SenD−* (*Varroa*-parasitised-brood-odour insensitive drone) or *SenD*+ (*Varroa*-parasitised-brood-odour sensitive drone). The data was analysed using a Generalised linear mixed model with group *ConQ × SenD*− as a reference. The course of observation and the drones’ origin (VSH-selected or nonselected line) were considered as factors in the analysis. The queen mother’s affiliation to one of the two lines (VSH-selected or nonselected line) was also included as a random factor in the model.

Group *SelQ × SenD−* exhibited the highest number of VSH-active bees in the two categories—beginner (7.8%) and helper (11.2%). Compared to the reference group, these results were statistically significant—beginner (GLMM, p < 0.001; CI 0.84; 1.64) and helper (GLMM, p < 0.001; CI 0.85; 1.54). The odds of *SelQ × SenD−* uncapping a parasitised cell were 3.5 times higher than that of the reference group (GLMM, OR 3.46; CI 2.32; 5.15).

Group *SelQ × SenD−* was followed by group *SelQ × SenD*+ (beginner: 3.4%, helper: 6.7%) (Table 1). Group *SelQ × SenD*+ displayed slightly but not significantly higher uncapping activity than the reference group (GLMM, p = 0.225; CI − 0.16; 0.68). The odds of this group initiating the uncapping of a parasitised cell were similar to those of the reference group.

**Table 1:** Grouping considering queen’s origin and drone’s olfactory sensitivity.

Group	Queens	Offspring	Beginner		Helper	
	N	N	N	%	N	%
*SelQ × SenD*+	12	1382	47	3.4	92	6.7
*SelQ × SenD−*	9	850	66	7.8	95	11.2
*ConQ × SenD*+	8	1273	18	1.4	32	2.5
*ConQ × SenD−*	11	1567	39	2.5	58	3.7

Summary of the number (N) of inseminated queens per group and their offspring (worker bees). Displayed are furthermore the number of beginner and helper bees in each group (N) and the corresponding equivalent in percent per group (%).

**Figure 3:**
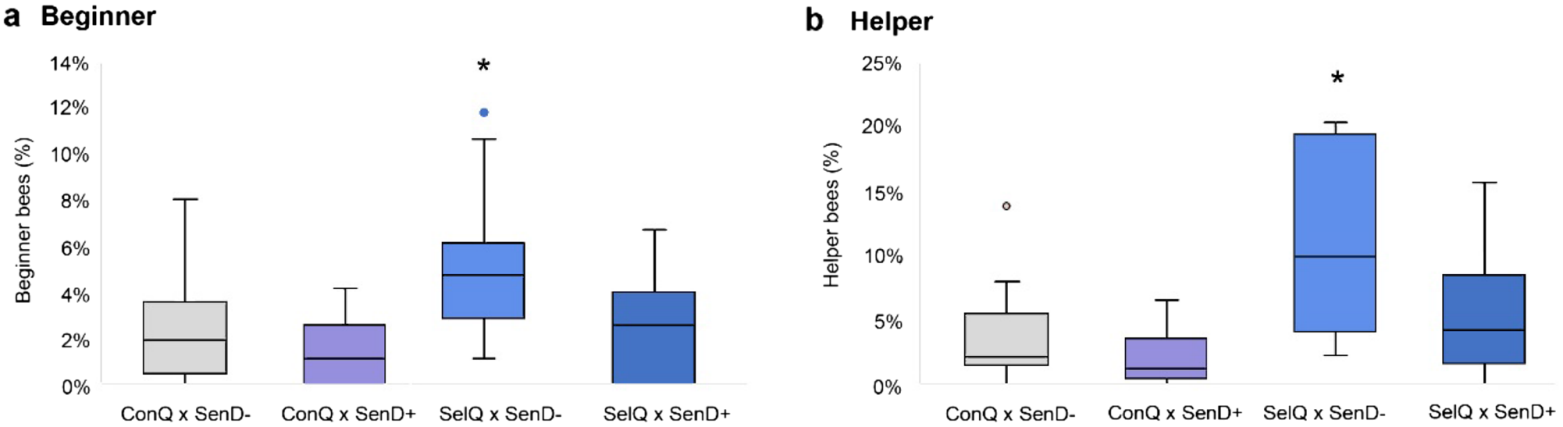
Boxplot of beginner and helper bees in groups based on the drones’ olfactory sensitivity. Displayed are median, standard deviation and outliers for the beginner (**a**) and helper (**b**) categories for each group. The proportions of beginner (**a**) and helper (**b**) bees during the three courses of the video observation experiment are displayed for each group. One colony had 11.8% beginner bees (outlier—group *SelQ × SenD−*). One colony exhibited 13.8% helper bees (outlier—group *ConQ × SenD−*). The number of colonies tested per group was as follows: 11 (*ConQ × SenD−*), 8 (*ConQ × SenD*+), 9 (*SelQ × SenD−*) and 12 (*SelQ × SenD*+). *Proportion of beginner and helper bees at the level of 0.001 significantly higher than reference group *ConQ × SenD−* (grey colour).

Group *ConQ × SenD*+ did not perform better than the reference group in any of the activities (see Suppl. Tables 2 and 3). In fact, the reference group exhibited more beginner (2.5%) and helper bees (3.7%) than the *ConQ × SenD*+ group (beginner: 1.4%, helper: 2.5%) (Fig. 3).

The origin of the queen mothers had significant effect on the beginner bees’ activity—GLMM, p < 0.001 (CI: 0.62; 1.47). The origin of the father drone played a significant effect on the helper bees’ activity with VSH-selected line drones producing more active offspring—GLMM, p < 0.001; CI 0.39; 0.95.

The three observation courses also exhibited differences in the number of active beginner and helper bees. The worker bees scored significantly higher in their beginner actions in courses two (GLMM, p = 0.017; CI 0.09; 0.97) and three (GLMM, p < 0.001; CI 0.58; 1.36) than the reference in course one. Course three also exhibited the highest results for helper activity (GLMM, p = 0.02; CI 0.05; 0.57).

#### VSH in groups considering the genetic origin

In the second evaluation step, the genetic origin of the queens (*SelQ, ConQ*) and drones (*SelD, ConD*) was used to restructure the aforementioned groups. The colonies participating in the experiment were divided into four new groups (Table 2 and Suppl. Table 4)—*ConQ × ConD*, *ConQ × SelD*, *SelQ × ConD*, *SelQ × SelD*. Group *ConQ × ConD* was used as reference group.

**Table 2:** Grouping considering genetic origin of queens and drones.

Group	Queens	Offspring	Beginner		Helper	
	N	N	N	%	N	%
*SelQ × SelD*	11	1076	59	5.5	112	10.4
*SelQ × ConD*	10	1156	54	4.7	75	6.5
*ConQ × SelD*	9	1384	35	2.5	65	4.7
*ConQ × ConD*	9	1456	22	1.5	25	1.7

Summary of the number (N) of inseminated queens per group and their offspring (worker bees). Displayed are furthermore the number of beginner and helper bees in each group (N) and the corresponding equivalent in percent per group (%).

**Figure 4:**
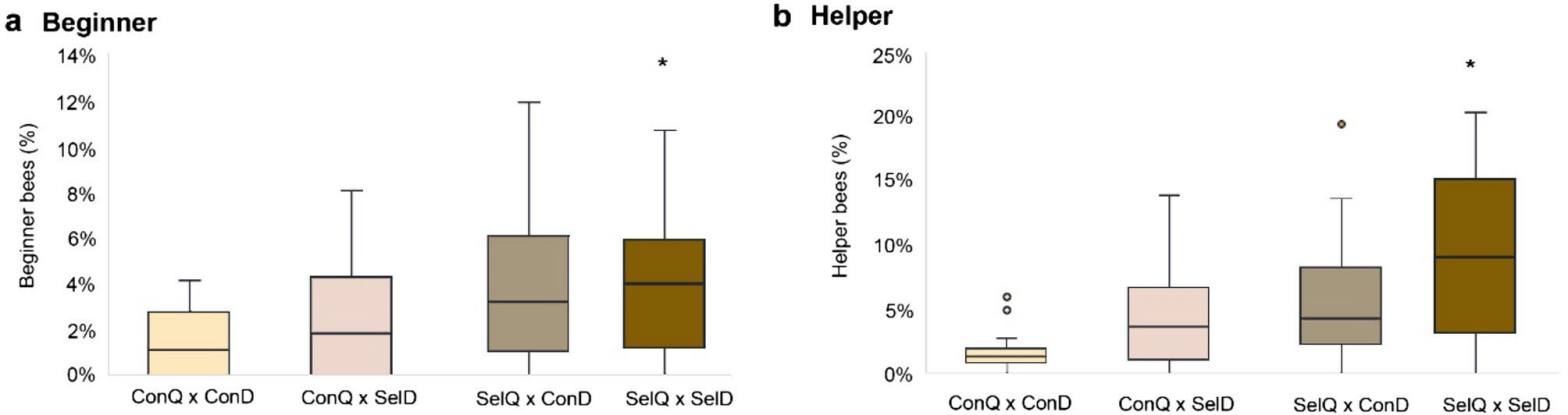
Boxplot of beginner and helper bees in groups based on the genetic origin of the queens and drones. Displayed are median, standard deviation and outliers for the beginner (**a**) and helper (**b**) categories for each group. The proportion of beginner (**a**) and helper (**b**) bees during the three courses of the video observation experiment are displayed for each group. One colony exhibited 19.4% helper bees (outlier—group *SelQ *×* ConD*). The number of colonies tested per group was as follows: 9 (*ConQ *×* ConD),* 9 (*ConQ *×* SelD*), 10 (*SelQ *×* ConD*) and 11 (*SelQ *×* SelD*). *Proportion of beginner and helper bees at the level of 0.001 significantly higher than reference group *ConQ *×* ConD* (beige colour).

When comparing the groups' beginner and helper activities to those of the reference group *ConQ × ConD*, a significant increase from the nonselected to VSH-selected line was observed. The pairing of queens from the VSH-selected line (*SelQ)* with drones from the VSH-selected line (*SelD*) delivered the highest number of active beginner (5.5%) and helper bees (10.4%) (Fig. 4). The results were statistically higher than those of the reference group *ConQ × ConD* (beginner: GLMM, p < 0.001; CI 1.20; 2.21; and helper: GLMM, p < 0.001; CI 1.47; 2.53). The odds of group *SelQ × SelD* uncapping a parasitised cell were 5.5 times higher than those of the reference group (GLMM, OR = 5.5; CI 3.31; 9.09).

The second highest results were achieved when inseminating a queen from the VSH-selected line (*SelQ*) with sperm from drones coming from the nonselected line (*ConD*). Group *SelQ *×* ConD* showed the second highest activity (beginner: 4.7%, helper: 6.5%). This group performed significantly better than the reference group *ConQ *×* ConD* in both activity categories (beginner: GLMM, p = 0.005; CI 0.44; 1.15; and helper: GLMM, p < 0.001; CI 0.81; 1.93). The results are listed in detail in Suppl. Tables 5 and 6. The odds of this group uncapping a parasitised cell were 2.7 times higher (GLMM, OR = 2.7; CI 1.55; 4.66) than those of the reference group.

Group *ConQ *×* SelD* exhibited significantly higher performance than the reference group in the helper activity (GLMM, p = 0.003; CI 0.53; 1.68). While the worker bees' performance in the beginner category was higher than that of the reference group, the results were not significant (GLMM, p = 0.18; CI – 0.24; 1.01).

The experimental course had no significant effect on the performance of the worker bees.

### Control cells

To check the specificity of the VSH-behaviour, each test course contained five control cells. These cells were opened and resealed without being infested with a mite to consider the possibility that the workers only responded to the manipulation of the cell cap. In the first round of observation, none of the control cells was opened by the worker bees during the video observation. These cells were *Varroa*-free. During the second round, the brood from one cell was removed. The other four cells were *Varroa*-free. In the last round, one cell contained a single nonfertile mite; the other four were not parasitised.

## Discussion

In the present study, 40 queens were each inseminated with sperm from one drone. A total of 5072 worker bees from the F1 generation were individually examined for their VSH. The aim of this multistage experiment was to assess the link between VSH and the drone's olfactory sensitivity, observed through conditioning the drones to an extract of *Varroa*-parasitised brood.

To our knowledge, this is the first conditioning experiment with drones using an extract from *Varroa*-parasitised brood. Compared to Chakroborty et al.^42^ who used live parasitised pupae as a conditioning stimulus, the extracts used in this experiment were much less concentrated. For *extract-low*, our goal was to reach the threshold of perception for the *Varroa*-parasitised-brood extract and select the most sensitive drones. *Extract-high* had a concentration almost twice as high as *extract-low* and served the purpose of selecting for drones unable to perceive the *Varroa*-parasitised-brood odour. Although the chosen experimental setup does not provide proof that drones could perceive the difference between healthy and parasitised brood, it shows their ability to perceive the complex odour bouquet of *Varroa*-parasitised brood at a very low concentration. Masterman et al*.*^41^ observed a difference in the discrimination abilities of hygienic and nonhygienic worker bees for brood odours. However, this does not seem to apply to drones. In contrast to worker bees, drone origin had no effect on their ability to perceive the CS+ during our experiment. Furthermore, the results from the PER conditioning experiment did not deliver any advantage to the F1 generation.

The drones' olfactory sensitivity to *extract-low* was not represented in the VSH of the drones' offspring. Moreover, the group with the highest results contained the sperm of drones that were insensitive to the *Varroa*-parasitised-brood odour (*SelQ *×* SenD−*).

When mated with queens from the VSH-selected line, the *Varroa*-parasitised-brood-odour sensitive drones produced colonies with more active beginner and helper bees than did the reference group *ConQ *×* SenD−*. However, those results were significant only for the helper activity (GLMM, p < 0.001; CI 0.39; 0.95). Provided that the single drone's perception ability is crucial for the manifestation of VSH in the next generation, we would have expected groups *SelQ *×* SenD*+ and *ConQ *×* SenD*+ to exhibit the highest activity in the observation. Contrary to our hypothesis, the *SelQ *×* SenD−* group produced the most active offspring in the three repetitions of the experiment. Furthermore, the *ConQ *×* SenD*+ group scored lower than the reference group, although the differences were not significant. Thus, our assumption that the negative conditioning outcome from the experiments with *extract-high* would be a reliable exclusion criterion, was incorrect.

There may be various reasons behind the inability of the conditioning experiment to ensure higher VSH activity in the next generation. The individual drones’ sensitivity to sucrose at the time of the experiment might have been different. Pankiw et al.^57^ described handling stress as one of the factors responsible for differences in sucrose sensitivity. From our observations, drones proved to be much more sensitive to conditioning length and weather conditions than worker bees. We observed a greater unwillingness of drones to respond to the CS+ and the sugar solution on cold or rainy days, although the temperature in the laboratory was regulated. Our observations corroborate earlier research conducted on drones^58,59^. Benatar et al.^58^ deemed the usual protocols used on worker bees unfit for drones. During our preliminary tests, we also observed high drone mortality if drones were treated according to existing bee protocols. Vareschi^59^ described differences between worker bee and drone conditioning, stating that drones are more "nervous" than worker bees. We, too, observed such a tendency. Throughout the experiment, we ensured the same nursing conditions for all test subjects through the drones' collective upbringing in one hive. We strived to ensure that the laboratory conditions were as uniform as possible. The number of trials was modified from eight to six to keep the drones as fit as possible for insemination. Nevertheless, the stress tolerance threshold of each individual differs^60^ and is a factor that is difficult to measure.

Another reason for the unsuccessful phenotyping of the drones through conditioning might be the strong sex dimorphism in the olfactory system of eusocial insects such as honeybees^55^. While queens and drones specialize in behavioural tasks such as mating, workers have a more diverse task range. Such specialization is also typical for other species, such as moths^61,62^, bark beetles^63^, cockroaches^64^, and ants^65,66^. The differences between both sexes encompass all stages of the olfactory pathway. The antennae of drones and workers exhibit sex-specific molecular specialization^67,68^. Drone antennae have a higher number of sensory cells (~ 339,000) than worker bees (~ 65,000)^69^. Of these, only one type—the so-called placoid sensilla—is present in large numbers in the drone's antennae, while the other types are either diminished in numbers or completely missing^55^. Most of the receptors on the drone's antennae are connected to the perception of the queen pheromone 9-ODA. Workers, on the other hand, exhibit receptors connected to pheromone communication, cuticular hydrocarbon perception and distinction of floral odours^68^.

Different epigenetic mechanisms, such as DNA methylation and histone posttranslational modifications, regulate the expression of receptor genes^70^. Kucharski et al*.*^71^ examined the expression of one odourant binding protein (OBP) gene—*obp11*—on the antennae of workers. OBP11 is also found in the *sensilla basiconica* of female ants^72^. It is involved in the accurate perception of cuticular hydrocarbons and pheromones, enabling workers to interact with each other and fulfil their social duties. While *obp11* is expressed in worker bee *sensilla basiconica*, it is silenced through methylation on drone antennae^71^.

According to Arnold et al*.*^73^, a well-pronounced sexual dimorphism in the glomeruli of the antennal lobe can be observed between worker bees and drones. While worker bees display only two structural types of glomeruli, drones exhibit a third glomerulus type, which is hypertrophied and responsible for the detection of queen pheromones^74^. Plant odours, on the other hand, are processed in the ordinary glomeruli of the antennal lobe^74^. While we proved that drones could perceive the extract used in our experiment, this ability is probably as unimportant to the drone's mating success as the distinction between two floral odours. It is therefore possible that the drone's ability to sense the odour of brood parasitised by *V. destructor* per se is of no advantage for the improvement of VSH. Moreover, the genes that are silenced in drones and cannot be measured by conditioning most likely play a larger role in the enhancement of VSH. If that is the case, odour conditioning would be unsuitable for detecting the best drones for breeding purposes.

The conditioning experiment might have also selected drones solely based on their better or worse learning abilities^58^. To rule out this possibility, we selected sensitive drones not only based on the results of the unrewarded tests but also on their whole performance during the experiment. Only drones that perceived the odour and distinguished it correctly from the CS- every time during the last trials and the unrewarded tests were chosen for insemination. While we acknowledge that the performed conditioning has some limitations for the achievement of our goal, we are optimistic regarding the potential of PER conditioning as a means for phenotyping drone olfactory sensitivity. Phenotyping in relation to an odour that is very easy to perceive for drones opens up the possibility of indirectly recognizing their general odour sensitivity. If used for breeding, this trait could lead to an increase in odour sensitivity in the drone’s female offspring towards *Varroa*-parasitised brood. Through an optimization of the exclusion criteria and the choice of another odour in a low concentration—for example 9-ODA—it might be possible to better select for odour sensitivity in the drone and pass on this trait to the next generation.

Our experiments also provide new information on the inheritance of VSH. When the group results were analysed with the genetic origin in mind, the number of beginner and helper actions increased when drones and/or queens of the VSH-selected line were used. The origin of the queen proved to play an even larger role than that of the drone. This observation was in accord with the substantial effect of the queen’s origin (VSH-selected/nonselected line) on the beginner activity when the results were analysed based on the PER conditioning experiment. The *Sel* queens produced offspring with a higher VSH activity when inseminated with sperm from *Con* drones than did *Con* queens inseminated with sperm from *Sel* drones. The odds of commencing a beginner activity compared to the reference group were as follows: 1.5-times higher for *ConQ × SelD* (OR; CI 0.79; 2.73), 2.7-times higher for *SelQ × ConD* (OR; CI 1.55; 4.66), and 5.5-times higher for *SelQ × SelD* (OR; CI 3.31; 9.09). The same tendency was observed for the helper activity: 3-times higher than the reference group for *ConQ × SelD* (OR; CI 1.71; 5.38), 3.9-times higher for *SelQ × ConD* (OR; CI 2.24; 6.86) and 7.4-times higher for *SelQ × SelD* (OR; CI 4.33; 12.53). These results lead us to believe that maternal effects play a significant role in the manifestation of VSH. Maternal effects shape behaviour and help offspring better adapt to changes in the environment. Maternal effects have been observed in many species^75–78^, including honeybees. Dloniak, French and Holekamp^78^ described rank-related maternal effects on offspring phenotype in spotted hyenas (*Crocuta crocuta*). Dominant females exhibited higher androgen concentrations in late pregnancy, which shaped the behaviour and social structure of the new generation. Storm and Lima^79^ described an "adaptive transgenerational maternal effect on offspring antipredator behaviour" in crickets. The offspring of mothers exposed to *Hogna helluo* spiders survived longer than the offspring of naive mothers. The forewarned crickets exhibited a behavioural change that manifested in a mobility reduction. Such behavioural changes have also been described in bees. Unger and Guzmán-Novoa^80^ experimented with crossbreeding of highly hygienic Russian bee strains and less hygienic Ontario bee strains. The hybrid bees with a "hygienic mother" and "control father" exhibited higher results for individual bees uncapping cells as well as removing the brood. On the other hand, "control queens" and "hygienic drones" produced an F1 generation with weaker hygienic behaviour. Spivak and Reuter^81^ assessed colonies with queens from a VSH-selected line naturally mated with unselected drones. Compared to unselected colonies, the hygienic colonies displayed a reduced mite load. Our findings further strengthen these observations.

This research demonstrates drones' ability to perceive low concentrations of brood-emitted odours. PER-conditioning with the selection criteria used in this experimental setting proved unsuitable for the enhancement of VSH. While an additive genetic effect was observed when drones from the VSH-selected line were paired with queens from the VSH-selected line, there was a tendency for maternal effects to also play an important role. Since both sexes inherit the same genes from their mother, it would be a big step towards creating a breeding strategy against *V. destructor* if a worker bee’s odour sensitivity could be measured on the haploid father’s side. Workers’ odour sensitivity towards parasitised brood is the key factor in *Varroa*-resistance. Therefore, further research is necessary to identify odours and suitable test methods to phenotype drones' odour sensitivity. If the heritability of such test results is sufficient, VSH can be improved more efficiently by the use of such individually tested drones in breeding.

## Materials and methods

### Extract preparation

An extract from *Varroa*-parasitised brood was created to mimic the complex composition of the distress signals emitted by the parasitised brood. A total of 190 mites were collected from a *Varroa*-infested colony at our institute. A brood frame with newly capped brood from a *Varroa-*free colony was chosen. The cell caps were cut open and lifted on one side using a razor blade. Only brood cells containing prepupae (9–10 days old) were infected. In each brood cell four mites were inserted using a moistened brush. The caps were subsequently resealed. The location of the parasitised cells was marked on translucent projector foil. The brood frame was placed back into the hive for two hours for the small incisions on the cell caps to be sealed by the nursing bees. After that, the frame was kept in an incubator for four days.

After that time, the parasitised pupae were extracted from the brood cells without damage. During the preparation process, the pupae were stored in an incubator at 35 °C on damp filter paper. Isopropanol was used as the base for the extract. The pupae were washed in 4 ml isopropanol for 10 min. The supernatant was decanted in special 2 ml glass vials with PVC lids and stored at − 20 °C. Two extracts with different concentrations were produced for this experiment—one extract obtained from 15 pupae (*extract-low*) and one from 25 pupae (*extract-high*).

### Testing for odour sensitivity

Having the process of localizing and uncapping parasitised brood cells in mind, we decided to present the odours in a manner that would allow direct contact with the stimulus and ensure that non-volatile chemicals such as oleic acid, the brood ester pheromone and tritriacontane are perceived^36,82–84^. We chose filter paper as a medium that was presented with the help of tweezers.

During the olfactory conditioning experiment, the solvent isopropanol—used during the preparation of the two extracts—was chosen as a CS−. As isopropanol was present in both the CS+ and the CS−, only drones that perceived the solved brood components sensed the difference between the two stimuli. If this were not the case, we expected that insensitive drones would show similar proboscis extension rates to both the CS+ and CS−.

Two PER conditioning experiments were carried out for the selection of drones that were to be used for artificial insemination:

1. Selecting *Varroa*-parasitised-brood-odour sensitive drones: 5 µl *extract-low* (see above) as the positive stimulus CS+ and 5 µl isopropanol as the negative stimulus CS−.
2. Selecting *Varroa*-parasitised-brood-odour insensitive drones: 5 µl *extract-high* (see above) as the positive stimulus CS+ and 5 µl isopropanol as the negative stimulus CS−.

For the conditioning experiments, eight colonies were chosen, and 100 newly hatched drones per origin were marked on the dorsal thorax with a chip. The drones were placed in a nursing hive with an unmated queen. Four of the chosen colonies came from a line selected for VSH, and the other four were of a nonselected line. A queen excluder was used to prevent drones from leaving the hive. After the drones reached reproductive age (14 days), the conditioning experiments were started.

The drones were collected from the hive shortly before the start of each conditioning and strapped in small metal tubes with paraffin tape. The immobilized drones were kept in a rack with numbered slots. A 50% sugar solution was used for the experiments. Only the drones that readily stretched their proboscis during the presentation of the sugar solution were used in the experiment.

The drones were presented with plain filter paper three times before the beginning of odour conditioning. This was done to prevent proboscis extension solely due to mechanical irritation from the filter paper. Each conditioning group consisted of eight drones. We aimed to equally represent every origin in these groups. Two conditioning experiments were conducted daily—one with each of the extracts. The chronological order of the tests (conditioning for sensitive drones, conditioning for insensitive drones) was changed each day to eliminate any bias due to the time of day.

During extensive preliminary experiments, we observed a decrease in drone reactions and difficulty collecting sperm after long-lasting conditioning experiments. Therefore, we modified the trial sequence of the conditioning described by Matsumoto et al.^46^ to shorten the experimental time.

The modified conditioning consisted of six trials with a specified order of stimuli presentation: CS+, CS−, CS−, CS+, CS+, CS−. The CS+ was enhanced by the administration of an unconditioned stimulus (US) in the form of a sugar solution. This was done with the help of a toothpick. The CS− was not reinforced. Each CS lasted 6 s. During the CS+ trials, the US was applied during the last 3 s of CS+ presentation. The intertrial interval was 5 min. No unpaired conditioning or exchange of the odours (isopropanol as CS+ and brood extract as CS−) was performed, as it was considered unnecessary for the achievement of our goals. The conditioning was used solely as a means of testing for odour perception and not to analyse learning behaviour.

The conditioning success was subsequently examined and recorded by a presentation of the two stimuli without the reward.

The following drones were considered for artificial insemination:

1. *Varroa*-parasitised-brood-odour sensitive drones displayed excellent odour perception of *extract-low* (15 pupae extract) by responding to the CS+ but not the CS− during the last two trials and during the unrewarded tests. (Trials: CS+, CS−, CS−, CS+, CS +, CS−; Unrewarded test: CS+, CS−).
2. *Varroa*-parasitised-brood-odour insensitive drones responded to the US throughout the experiment with *extract-high* (25 pupae) but showed no positive responses to the CS+ during the last two trials, and the unrewarded tests indicated a negative conditioning outcome and the inability to perceive the extract of *Varroa*-parasitised pupae.

A total of 223 drones were tested with *extract-low*, while 202 drones were assessed using *extract-high*. Drones that stretched their proboscis at the first presentation of the CS+ were excluded as well as those that stopped responding to the stimulus during the experiment. The number of excluded drones amounted to 22% for *extract-low* and 16% for *extract-high*.

### Artificial insemination

The drones were brought back to the hive after each conditioning for recovery before the sperm were extracted. Sperm extraction took place immediately before insemination^85^.

**Figure 5:**
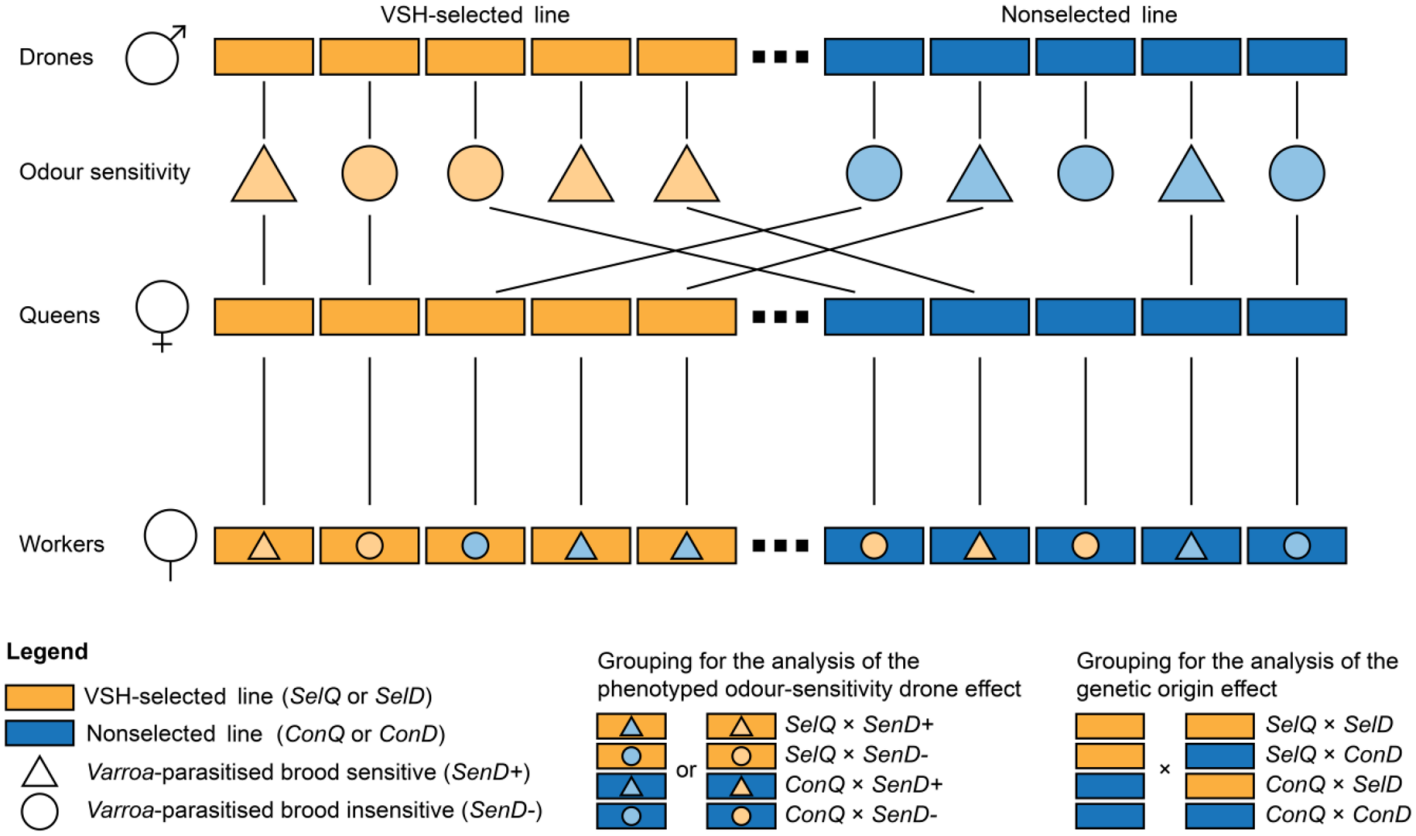
Mating scheme used during one-drone insemination. Drones from each of the two lines—VSH-selected line (*SelD*, yellow colour) and nonselected line (*ConD,* blue colour)—were tested for their odour sensitivity towards the extract of *Varroa*-parasitised brood. Drones, which perceived the *Varroa*-parasitised-brood-odour were referred to as “*Varroa*-parasitised-brood-odour sensitive" (*SenD*+) and marked with a triangle. Drones, that did not perceive the odour were referred to as “*Varroa*-parasitised-brood-odour insensitive” (*SenD−*) and marked with a circle. The tested drones were subsequently used for the insemination of queens from both VSH-selected (*SelQ*, yellow colour) and nonselected line (*ConQ*, blue colour). The offspring workers were placed into four groups considering the queen's genetic origin and the drone's olfactory sensitivity towards the *Varroa*-parasitised-brood extract. The workers were subsequently assessed for their VSH in a video observation test.

The queens originated from lines selected for their hygienic behaviour towards *V. destructor* (VSH-selected line) and from institute-owned lines (nonselected line). The one-drone insemination was conducted using the mating scheme displayed in Fig. 5.

Of a total of 50 queens, 40 took part in the experiment. The rest did not produce enough eggs in time for the video observations or died. The inseminated queens were housed in mini nucleus hives (Segeberger®) with young bees. All mini nucleus hives were located on the institute terrain in close proximity to one another. The mini nucleus hives were fitted with two food frames each (honey and pollen) and two brood frames. The worker bees for the mini nucleus hives came from colonies kept in the institute, especially for the purpose of queen rearing. Each mini nucleus hive received approximately the same number of worker bees. The worker bees were supplied with feed dough to ensure adequate food storage. The flight hole was narrowed to prevent possible robbing behaviour. Once all the inseminated queens had started laying eggs, each mini nucleus hive received an empty brood frame at the same time to ensure that all the bees for the infrared video observation were of the same age.

After the young bees hatched, they were collected daily within a week and marked individually with a numbered plate on the dorsal thorax. Afterwards, they were placed in the video surveillance unit described by Bienefeld et al*.*^37^. A *Varroa-*free brood frame with freshly capped brood was taken from an institute-owned hive, and 60 brood cells were infected with one mite each. Five control cells were opened and resealed without being artificially infested. The brood frame was placed in the observation unit, and the recording was started.

For six days, bee activity was monitored using an infrared camera. The video recording analysis was carried out manually with the help of a software program—Beehaviour—specially created for this purpose (Batz et al*.*, submitted).

### Statistical analysis

#### Analysis of PER conditioning experiment

The drones were split into two groups for the statistical analysis, considering their origin (VSH-selected line/nonselected line). Acquisition curves were plotted in addition to the analysis.

The outcome (0—unsuccessful, 1—successful) of the unrewarded tests was examined using a binomial generalized linear mixed model (GLMM) with a logit function in SPSS V. 25. The alpha-level was set at 0.05. The drones coming from the control line were set as a reference group by the model. The stimulus effect (reinforced, non-reinforced) was also considered. The temperature during the experiment and the mother of each drone were both set as random factors.

#### Video-observation analysis

While observing the VSH recordings of the drones’ offspring, two activities were used to evaluate the VSH of the new generation. The beginner activity was defined by the first worker opening an infested cell and the helper activity—the workers that enlarged the hole after the beginner had created it. If the cell caps were opened and resealed multiple times, the new beginner and helper bees were written down. One course of video observation was completed in year one. In the second year, two courses of video observations were performed. A total of 5072 bees were recorded during the experiment: 1694 in course one, 1696 in course two and 1682 in course three. More detailed information on the composition of each group and the number of worker bees is described in Suppl. Tables 7 and 8.

#### VSH of groups considering the conditioning outcome

The video recording results were analysed through a binomial GLMM with a logit function in SPSS V.25.

Group *ConQ × SenD−* was used as a reference. The courses of observation—one, two or three—and the drone's origin (VSH-selected line, nonselected line) were considered fixed effects. By including the drone's origin in the regression, the model provided more accurate insight into the PER conditioning and its explanatory power for the results. Course one and nonselected lines were chosen as reference values. The individual effect of each queen mother on the VSH of her offspring was set as a random factor in the regression model.

#### VSH of groups considering the parental origin

In a second step, the video observation results were analysed with consideration of the parental origin of queens and drones and ignoring the PER conditioning results. The statistical analysis was conducted using a binomial GLMM with a logit function. Group *ConQ × ConD* was set as the reference group. The course of observation was again considered a fixed effect. Course one was set as a reference.

## Supplementary Information

Supplementary Information.

## Data Availability

The datasets generated and analysed during the current study are available from the corresponding author on reasonable request.
