# Supplementary material for: Suitability of drone olfactory sensitivity as a selection trait for Varroa-resistance in honeybees
Source: Sci Rep. 2021 Sep 6;11:17703. doi: 10.1038/s41598-021-97191-w (PMC8421409; doi:10.1038/s41598-021-97191-w)
Supplement: Supplementary file 1 — Supplementary Information. [file 41598_2021_97191_MOESM1_ESM.docx]

Suitability of drone olfactory sensitivity as a selection trait for *Varroa*-resistance in honeybees

Ivelina Ivanova* ^1, 2^, Kaspar Bienefeld ^1^

^1^ Institute for Bee Research Hohen Neuendorf, Friedrich-Engels-Str. 32, 16540 Hohen Neuendorf, Germany

^2^ Institute of Parasitology and Tropical Veterinary Medicine, Faculty of Veterinary Medicine, Free University of Berlin, Robert-von-Ostertag-Str. 7-13, Building 35, 14163 Berlin, Germany

*i.ivanova@fu-berlin.de

Supplementary Materials

| **Group** | | **Drones from VSH-selected line (%)** | **Drones from nonselected line (%)** |
| --- | --- | --- | --- |
| *Varroa*-parasitised-brood odour insensitive drones | ***ConQ x SenD-*** | 64 | 36 |
|  | ***SelQ x SenD-*** | 67 | 33 |
| *Varroa*-parasitised-brood odour sensitive drones | ***ConQ x SenD+*** | 38 | 62 |
|  | ***SelQ x SenD+*** | 42 | 58 |

Supplementary Table 1 – Summary of drones used in the one-drone insemination. Displayed are the proportions of drones from the VSH-selected and the nonselected line in each group. The insemination was conducted without regards to the genetic origin of the drones.

| **Statistical analysis of beginner activity considering drones olfactory sensitivity (GLMM)** | | | | | | |
| --- | --- | --- | --- | --- | --- | --- |
| **Groups** | Reg.-Coeff. | S.E. | Sig. | 95% CI | OR | 95% CI(OR) |
| **Intercept** | -4.34 | 0.23 | 0.001 | -4.78; -3.90 | 0.01 | 0.01; 0.02 |
| ***SelQ x SenD+*** | 0.26 | 0.21 | 0.225 | -0.16; 0.68 | 1.29 | 0.85; 1.96 |
| ***SelQ x SenD-*** | 1.24 | 0.20 | 0.001 | 0.84; 1.64 | 3.46 | 2.32; 5.15 |
| ***ConQ x SenD+*** | -0.42 | 0.28 | 0.137 | -0.98; 0.13 | 0.66 | 0.38; 1.14 |
| ***ConQ x SenD-*** | 0^a^ | - | - | - | - | - |
|  |  |  |  |  |  |  |
| ***Course 3*** | 0.97 | 0.20 | 0.001 | 0.58; 1.36 | 2.64 | 1.79; 3.89 |
| ***Course 2*** | 0.53 | 0.22 | 0.017 | 0.09; 0.97 | 1.70 | 1.10; 2.64 |
| ***Course 1*** | 0^a^ | - | - | - | - | - |
|  |  |  |  |  |  |  |
| ***SelD*** | 0.17 | 0.17 | 0.313 | -0.16; 0.49 | 1.18 | 0.85; 1.64 |
| ***ConD*** | 0^a^ | - | - | - | - | - |
| ^a^ – reference group | | | | | | |

Supplementary Table 2– Statistical analysis of beginner activity considering drones olfactory sensitivity. Group *ConQ x SenD-* , course 1 and ConD are chosen as reference groups by the model. Displayed are the differences in beginner activity between the four groups *ConQ x SenD-, ConQ x SenD+, SelQ x SenD-, SelQ x SenD+*. The three courses of observation are also listed, as well as the effect of the drones origin (VSH-selected line, nonselected line) on the offspring’s uncapping activity. The significance level lies at p<0.05.

| **Statistical analysis of helper activity considering drones olfactory sensitivity (GLMM)** | | | | | | |
| --- | --- | --- | --- | --- | --- | --- |
| **Groups** | Reg.-Coeff. | S.E. | Sig. | 95% CI | OR | 95% CI(OR) |
| **Intercept** | -3.85 | 0.17 | 0.001 | -4.21; -3.48 | 0.02 | 0.02; 0.03 |
| ***SelQ x SenD+*** | 0,76 | 0,17 | 0,001 | 0,40; 1,12 | 2,14 | 1,50; 3,06 |
| ***SelQ x SenD-*** | 1,19 | 0,16 | 0,001 | 0,85; 1,54 | 3,29 | 2,33; 4,65 |
| ***ConQ x SenD+*** | -0.18 | 0.22 | 0.417 | -0.64; 0.28 | 0.85 | 0.53; 1.32 |
| ***ConQ x SenD-*** | 0^a^ | - | - | - | - | - |
|  |  |  |  |  |  |  |
| ***Course 3*** | 0.41 | 0.14 | 0.008 | 0.12; 0.70 | 1.51 | 1.13; 2.01 |
| ***Course 2*** | 0.05 | 0.15 | 0.750 | -0.27; 0.37 | 1.05 | 0.76; 1.45 |
| ***Course 1*** | 0^a^ | - | - | - | - | - |
|  |  |  |  |  |  |  |
| ***SelD*** | 0.67 | 0.13 | 0.001 | 0.39; 0.95 | 1.96 | 1.48; 2.58 |
| ***ConD*** | 0^a^ | - | - | - | - | - |
| ^a^ – reference group | | | | | | |

Supplementary Table 3– Statistical analysis of helper activity considering drones olfactory sensitivity. Group *ConQ x SenD-* , course 1 and ConD are chosen as reference groups by the model. Displayed are the differences in helper activity between the four groups *ConQ x SenD-, ConQ x SenD+, SelQ x SenD-, SelQ x SenD+*. The three courses of observation are also listed, as well as the effect of the drones origin (VSH-selected line, nonselected line) on the offspring’s VSH activity. The significance level lies at p<0.05.

| **Colony ID** | **Group considering conditioning of drone** | **Group considering hygienic status of queen and drone** | **Average number beginner bees per colony (%)** | **Average number helper bees per colony (%)** |
| --- | --- | --- | --- | --- |
| 166 | ***ConQ x SenD+*** | ***ConQ x ConD*** | 3.0 | 6.9 |
| 168 | ***ConQ x SenD+*** | ***ConQ x ConD*** | 1.1 | 1.1 |
| 169 | ***ConQ x SenD-*** | ***ConQ x ConD*** | 3.2 | 3.2 |
| 291 | ***ConQ x SenD-*** | ***ConQ x ConD*** | 3.5 | 4.3 |
| 292 | ***ConQ x SenD-*** | ***ConQ x ConD*** | 4.9 | 3.9 |
| 293 | ***ConQ x SenD+*** | ***ConQ x ConD*** | 0.8 | 2.0 |
| 295 | ***ConQ x SenD+*** | ***ConQ x ConD*** | 7.1 | 6.0 |
| 518 | ***ConQ x SenD-*** | ***ConQ x ConD*** | 0.0 | 0.0 |
| 888 | ***ConQ x SenD+*** | ***ConQ x ConD*** | 1.1 | 1.1 |
| 170 | ***ConQ x SenD-*** | ***ConQ x SelD*** | 6.8 | 11.0 |
| 171 | ***ConQ x SenD-*** | ***ConQ x SelD*** | 6.1 | 6.1 |
| 290 | ***ConQ x SenD-*** | ***ConQ x SelD*** | 14.2 | 21.5 |
| 294 | ***ConQ x SenD+*** | ***ConQ x SelD*** | 2.0 | 3.1 |
| 488 | ***ConQ x SenD-*** | ***ConQ x SelD*** | 0.0 | 0.0 |
| 489 | ***ConQ x SenD-*** | ***ConQ x SelD*** | 1.1 | 1.1 |
| 516 | ***ConQ x SenD+*** | ***ConQ x SelD*** | 0.0 | 0.0 |
| 517 | ***ConQ x SenD-*** | ***ConQ x SelD*** | 0.0 | 7.9 |
| 519 | ***ConQ x SenD-*** | ***ConQ x SelD*** | 1.1 | 3.3 |
| 999 | ***ConQ x SenD+*** | ***ConQ x SelD*** | 6.7 | 6.4 |
| 90081 | ***SelQ x SenD-*** | ***SelQ x ConD*** | 11.8 | 19.4 |
| 90082 | ***SelQ x SenD+*** | ***SelQ x ConD*** | 4.4 | 7.2 |
| 90183 | ***SelQ x SenD+*** | ***SelQ x ConD*** | 9.0 | 7.7 |
| 90184 | ***SelQ x SenD+*** | ***SelQ x ConD*** | 5.3 | 13.3 |
| 90190 | ***SelQ x SenD+*** | ***SelQ x ConD*** | 6.7 | 13.5 |
| 90583 | ***SelQ x SenD+*** | ***SelQ x ConD*** | 0.0 | 2.4 |
| 90584 | ***SelQ x SenD-*** | ***SelQ x ConD*** | 5.6 | 2.2 |
| 90586 | ***SelQ x SenD+*** | ***SelQ x ConD*** | 1.1 | 3.4 |
| 90588 | ***SelQ x SenD-*** | ***SelQ x ConD*** | 3.1 | 7.4 |
| 90589 | ***SelQ x SenD+*** | ***SelQ x ConD*** | 0.0 | 0.0 |
| 90080 | ***SelQ x SenD+*** | ***SelQ x SelD*** | 6.7 | 15.6 |
| 90083 | ***SelQ x SenD-*** | ***SelQ x SelD*** | 4.7 | 9.4 |
| 90181 | ***SelQ x SenD+*** | ***SelQ x SelD*** | 8.0 | 6.0 |
| 90187 | ***SelQ x SenD-*** | ***SelQ x SelD*** | 9.9 | 19.2 |
| 90191 | ***SelQ x SenD-*** | ***SelQ x SelD*** | 1.1 | 2.2 |
| 90579 | ***SelQ x SenD+*** | ***SelQ x SelD*** | 0.0 | 9.0 |
| 90580 | ***SelQ x SenD-*** | ***SelQ x SelD*** | 1.3 | 0.0 |
| 90581 | ***SelQ x SenD-*** | ***SelQ x SelD*** | 10.6 | 20.2 |
| 90582 | ***SelQ x SenD+*** | ***SelQ x SelD*** | 0.0 | 4.0 |
| 90585 | ***SelQ x SenD-*** | ***SelQ x SelD*** | 5.7 | 10.3 |
| 90587 | ***SelQ x SenD-*** | ***SelQ x SelD*** | 2.8 | 19.4 |

Supplementary Table 4 – Colony numbers and their affiliation to the experimental groups. The table also displays the average number of beginner and helper bees for each colony in percent.

| **Statistical analysis of beginner activity considering parental genetic origin (GLMM)** | | | | | | |
| --- | --- | --- | --- | --- | --- | --- |
| **Groups** | Reg.-Coeff. | S.E. | Sig. | 95% CI | OR | 95% CI(OR) |
| **Intercept** | -3.83 | 0.21 | 0.001 | -4.35; -3.30 | 0.02 | 0.01; 0.04 |
| ***SelQ x SelD*** | 1.70 | 0.21 | 0.001 | 1.20; 2.21 | 5.48 | 3.31; 9.09 |
| ***SelQ x ConD*** | 0.99 | 0.23 | 0.005 | 0.44; 1.54 | 2.69 | 1.55; 4.66 |
| ***ConQ x SelD*** | 0.39 | 0.25 | 0.179 | -0.24; 1.01 | 1.47 | 0.79; 2.73 |
| ***ConQ x ConD*** | 0 ^a^ | - | - | - | - | - |
|  |  |  |  |  |  |  |
| ***Course 3*** | 0.06 | 0.15 | 0.709 | -0.30; 0.42 | 1.06 | 0.740; 1.52 |
| ***Course 2*** | -0.22 | 1.63 | 0.226 | -0.62; 0.18 | 0.80 | 0.54; 1.20 |
| ***Course 1*** | 0 ^a^ | - | - | - | - | - |
| ^a^ – reference group | | | | | | |

Supplementary Table 5- Statistical analysis of beginner activity considering genetic origin of the queen mother and the fathering drone. Displayed are differences in the beginner activity of the four groups – *ConQ x ConD, ConQ x SelD, SelQ x ConD, SelQ x SelD*. The three courses of observation are also listed. Course 1 and group *ConQ x ConD* are chosen as reference by the model. The significance level lies at p<0.05.

| **Statistical analysis of helper activity considering parental genetic origin (GLMM)** | | | | | | |
| --- | --- | --- | --- | --- | --- | --- |
| **Groups** | Reg.-Coeff. | S.E. | Sig. | 95% CI | OR | 95% CI(OR) |
| **Intercept** | -4.21 | 0.22 | 0.001 | -4.76; -3.67 | 0.02 | 0.01; 0.03 |
| ***SelQ x SelD*** | 2.00 | 0.22 | 0.001 | 1.47; 2.53 | 7.36 | 4.33; 12.53 |
| ***SelQ x ConD*** | 1.37 | 0.23 | 0.001 | 0.81; 1.93 | 3.92 | 2.24; 6.86 |
| ***ConQ x SelD*** | 1.11 | 0.23 | 0.003 | 0.53; 1.68 | 3.03 | 1.71; 5.38 |
| ***ConQ x ConD*** | 0 ^a^ | - | - | - | - | - |
|  |  |  |  |  |  |  |
| ***Course 3*** | 0.40 | 0.14 | 0.025 | 0.07; 0.74 | 1.50 | 1.07; 2.09 |
| ***Course 2*** | 0.07 | 0.15 | 0.685 | -0.31; 0.44 | 1.07 | 0.73; 1.55 |
| ***Course 1*** | 0 ^a^ | - | - | - | - | - |
| ^a^ – reference group | | | | | | |

Supplementary Table 6 – Statistical analysis of helper activity considering genetic origin of the queen mother and the fathering drone. Displayed are differences in the helper activity of the four groups – *ConQ x ConD, ConQ x SelD, SelQ x ConD, SelQ x SelD*. The three courses of observation are also listed. Course 1 and group *ConQ x ConD* are chosen as reference by the model. The significance level lies at p<0.05.

| **Group sizes considering queen’s origin and odour sensitivity of drones** | | | | |
| --- | --- | --- | --- | --- |
| **Crossbreed** | ***Varroa*-parasitised-brood-odour sensitive drone (*SenD+*)** | | ***Varroa*-parasitised-brood-odour insensitive drone (*SenD-)*** | |
|  |  | N |  | N |
| **Queen from VSH-selected line *(SelQ)*** | *SelQ* ***x*** *SenD+* | 1382 | *SelQ* ***x*** *SenD-* | 850 |
| **Queen from nonselected line *(ConQ)*** | *ConQ* ***x*** *SenD+* | 1273 | *ConQ* ***x*** *SenD -* | 1567 |

Supplementary Table 7 - Group sizes considering queen’s origin and odour sensitivity of drones. VSH-selected line queens (*SelQ*) and nonselected line queens (*ConQ*) were inseminated with sperm from drones, which participated in the PER conditioning. Drones deemed as sensitive to the *Varroa*-parasitised-brood odour were marked with *SenD+*. Those which were defined as insensitive were marked with *SenD*-. The number of offspring workers in each group participating in the video observation is listed next to the mating combination. N stands for the total number of bees in each group.

| **Group sizes considering queen and drone genetic origin** | | | | |
| --- | --- | --- | --- | --- |
| **Breeding line queen/breeding line drone** | **Drone from VSH-selected line (*SelD*)** | | **Drone from nonselected line (*ConD*)** | |
|  |  | N |  | N |
| **Queen from VSH-selected line *(SelQ)*** | *SelQ* ***x*** *SelD* | 1076 | *SelQ* ***x*** *ConD* | 1156 |
| **Queen from nonselected line *(ConQ)*** | *ConQ* ***x*** *SelD* | 1384 | *ConQ* ***x*** *ConD* | 1456 |

Supplementary Table 8 - Group sizes considering queen and drone genetic origin VSH-selected line queens (*SelQ*) and nonselected line queens (*ConQ*) were inseminated with sperm from VSH-selected line drones (*SelD*) and nonselected line drones (*ConD*) The number of offspring workers in each group participating in the video observation is listed next to the mating combination. N stands for the total number of bees in each group.
